# Biomarkers of Progression after HIV Acute/Early Infection: Nothing Compares to CD4^+^ T-cell Count?

**DOI:** 10.3390/v10010034

**Published:** 2018-01-13

**Authors:** Gabriela Turk, Yanina Ghiglione, Macarena Hormanstorfer, Natalia Laufer, Romina Coloccini, Jimena Salido, César Trifone, María Julia Ruiz, Juliana Falivene, María Pía Holgado, María Paula Caruso, María Inés Figueroa, Horacio Salomón, Luis D. Giavedoni, María de los Ángeles Pando, María Magdalena Gherardi, Roberto Daniel Rabinovich, Pedro A. Pury, Omar Sued

**Affiliations:** 1 CONICET-Universidad de Buenos Aires, Instituto de Investigaciones Biomédicas en Retrovirus y SIDA (INBIRS), Universidad de Buenos Aires- CONICET, Paraguay 2155 Piso 11, Buenos Aires C1121ABG, Argentina; yghiglione@fmed.uba.ar (Y.G.); nlaufer@fmed.uba.ar (N.L.); romina.coloccini@gmail.com (R.C.); jimenasalido@gmail.com (J.S.); trifonecesar@gmail.com (C.T.); mariajulia83@gmail.com (M.J.R.); juliana.falivene@gmail.com (J.F.); piaholgado@gmail.com (M.P.H.); pau_caruso@hotmail.com (M.P.C.); hsalomon@fmed.uba.ar (H.S.); mpando@fmed.uba.ar (M.A.P.); mgherardi@fmed.uba.ar (M.M.G.); rabinovichra@yahoo.com.ar (R.D.R.); 2 Fundación Huésped, Buenos Aires C1202ABB, Argentina; hormanstorferm@gmail.com (M.H.); maria.figueroa@huesped.org.ar (M.I.F.); omar.sued@huesped.org.ar (O.S.); 3 Hospital Juan A. Fernández, Unidad Enfermedades Infecciosas, Buenos Aires C1425AGP, Argentina; 4 Southwest National Primate Research Center, Texas Biomedical Research Institute, San Antonio, TX 78227, USA; lgiavedoni@txbiomed.org; 5 Facultad de Matemática, Astronomía, Física y Computación, Universidad Nacional de Córdoba, Córdoba X5000HUA, Argentina; pury@famaf.unc.edu.ar

**Keywords:** HIV, biomarkers, acute infection, disease progression, decision trees, soluble plasma factors, HLA, immune responses

## Abstract

Progression of HIV infection is variable among individuals, and definition disease progression biomarkers is still needed. Here, we aimed to categorize the predictive potential of several variables using feature selection methods and decision trees. A total of seventy-five treatment-naïve subjects were enrolled during acute/early HIV infection. CD4^+^ T-cell counts (CD4TC) and viral load (VL) levels were determined at enrollment and for one year. Immune activation, HIV-specific immune response, Human Leukocyte Antigen (HLA) and C-C chemokine receptor type 5 (CCR5) genotypes, and plasma levels of 39 cytokines were determined. Data were analyzed by machine learning and non-parametric methods. Variable hierarchization was performed by Weka correlation-based feature selection and J48 decision tree. Plasma interleukin (IL)-10, interferon gamma-induced protein (IP)-10, soluble IL-2 receptor alpha (sIL-2Rα) and tumor necrosis factor alpha (TNF-α) levels correlated directly with baseline VL, whereas IL-2, TNF-α, fibroblast growth factor (FGF)-2 and macrophage inflammatory protein (MIP)-1β correlated directly with CD4^+^ T-cell activation (*p* < 0.05). However, none of these cytokines had good predictive values to distinguish “progressors” from “non-progressors”. Similarly, immune activation, HIV-specific immune responses and HLA/CCR5 genotypes had low discrimination power. Baseline CD4TC was the most potent discerning variable with a cut-off of 438 cells/μL (accuracy = 0.93, κ-Cohen = 0.85). Limited discerning power of the other factors might be related to frequency, variability and/or sampling time. Future studies based on decision trees to identify biomarkers of post-treatment control are warrantied.

## 1. Introduction

Human Immunodeficiency Virus (HIV) infection causes an irreversible deterioration of the immune system ultimately leading to the development of AIDS in the vast majority of infected persons. Following virus transmission, acute/early phase of infection is characterized by a high-level peak of viremia, rapid loss of CD4^+^ T-cells in both peripheral blood and mucosal lymphoid tissues, and, in some cases, clinical symptoms [1,2]. Emergence of HIV-specific CD8^+^ T-cell response is associated with the drop of plasma viremia to a stable level; known as the viral set-point [3]. Within this general framework, it is also known that the rate of disease progression after acquiring the infection is very variable among individuals, allowing the identification of different subgroups: rapid progressors, typical progressors, viremic controllers or elite controllers [4].

The application of biomarkers in the diagnosis and management of cardiovascular diseases, several infections, immune and genetic disorders, as well as cancer is well known [5]. However, finding a reliable biomarker able to predict the rate of disease progression after acute/early HIV infection remains an important challenge. Nowadays, the HIV treatment guidelines recommended by the United States, the World Health Organization, the International AIDS Society and the European AIDS Clinical Society, adhere to providing universal combined antiretroviral treatment (cART) regardless of the infected persons were undergoing recent or chronic infection [6,7,8,9]. This might diminish the interest in finding a biomarker of disease progression. However, it should be considered that the application of biomarkers is beyond disease prediction and monitoring. Identifying biomarkers helps dissect the mechanisms underlying pathogenic processes and also plays an important role in drug discovery/design, development and validation [10]. On the other hand, differential public health approaches are necessary to maximize the use of constrained resources in low and middle-low income countries. Finally, even though the guidelines state that therapy should be initiated soon after diagnosis, they are not set in stone. Decisions must still be made on a case-by-case basis and the need to defer cART because of the presence of clinical and/or psychosocial factors. Therefore, biomarkers may aid in the medical decision for these particular cases. Thus, HIV biomarker is a field that still deserves continuous research to fill in the gaps in different aspects of HIV pathogenesis, discover new targets, improve current HIV treatment strategies, and aid in cure research.

Thus far, CD4^+^ T-cell counts and plasma viral load (VL) levels have remained the strongest correlates of progression and are the two markers routinely used in the clinical setting to monitor the infection [1]. In addition, other parameters such as levels of cell-associated viral DNA [11,12], immune activation and inflammation [13,14], several cytokines [15,16,17,18], HIV-specific immune responses [19,20,21,22,23,24,25], and genetic variants (recently reviewed in [26]) have been shown to be associated with disease progression and proposed as potential biomarkers. However, different limitations preclude them from being installed into the routine practice.

In 2008, an ongoing multicenter Argentine observational cohort of subjects diagnosed during primary HIV infection, named *Grupo Argentino de Seroconversión* study group, was created in Argentina. The aim of this consortium is to gather epidemiological, clinical, immunological and virological data of the individuals enrolled to dissect variables associated with different rates of disease progression soon after infection, and ultimately to identify potential markers associated with progression [27]. In an initial study based only on clinical variables, baseline VL > 100,000 copies/mL was associated with progression [27]. Later, in this cohort, it was demonstrated that CD8^+^ T-cell specificity (higher proportion of early anti-Group-specific antigen (Gag) T-cells), functionality (enhanced viral inhibitory activity) and phenotype (preserved differentiation and lower proportion of exhausted cells) correlated with delayed disease progression [28,29]. Moreover, disease progression, in terms of viral load, could be correlated with a genetic score built based on Human Leukocyte Antigen (HLA) and C-C chemokine receptor type 5 (CCR5) genotypes genotypes [30]. Finally, it could be demonstrated that higher baseline T helper 17 (Th17) cell percentages were associated with lower baseline levels of immune T-cell activation and to lower rates of disease progression [31]. 

In this study, we took advantage of all these variables comprehensively studied in our cohort and that have been individually associated with disease progression and aimed to categorize their predictive potential. The complete set included 88 variables (16 clinical variables, 10 immunological variables, 10 genetic variables and 52 variables related to soluble plasma factors) measured in up to 75 individuals. Due to the large number of variables analyzed, we first ranked sets of different variables based on their correlations with each class of progression. Then, using the top ranked variables, we studied their predictive power by constructing decision trees. This machine learning approach generates a hierarchy of variables automatically and provides a quantitative measure of the predictive capability of a given set. Moreover, the decision trees automatically provide the best cut-off values for continuous variables and they are reliable classifiers.

## 2. Materials and Methods

### 2.1. Study Subjects

Seventy-five subjects with ongoing acute/early primary HIV infection (PHI) were enrolled by the *Grupo Argentino de Seroconversión* study group [27], an ongoing multicenter Argentine observational cohort of subjects diagnosed during primary HIV infection. Inclusion criteria for enrolment in the cohort are: age >16 years at first evaluation, confirmed diagnosis of primary HIV infection, and first (baseline) medical and laboratory evaluation (i.e., CD4 cell count and plasma HIV RNA) within six months of the probable date of infection. Primary HIV infection is defined as: (1) detection of HIV RNA or p24 antigen with a simultaneous negative or indeterminate Western blot assay; or (2) positive Western blot with a negative diagnostic within the previous six months. Hence, it includes subjects up to Fiebig VI. Data included in this study were obtained from enrolled subjects while they were off-ART. Samples and data obtained after ART initiation were not considered in the analysis. Of note, most subjects were enrolled between years 2008 and 2012, before international [32] and national [33] treatment guidelines recommended that all HIV-infected subjects should initiate ART immediately after diagnosis. Additionally, samples from 21 HIV-seronegative healthy donors (HD) were obtained from voluntary blood donors at the *Sanatorio Dr Julio Mendez* blood bank (Buenos Aires, Argentina). All donors were between 18 and 65 years old; completed and passed a survey on blood donation; and were screened for serological markers of HIV, Hepatitis C virus, Hepatitis B virus, Human T- cell lymphotropic virus I and II, Syphilis, Chagas disease, and Brucellosis before being accepted as donors.

### 2.2. Ethical Considerations

This study was reviewed and approved by two institutional review boards (IRB): *Comité de Ética Humana, Facultad de Medicina, Universidad de Buenos Aires* (11/11/2010 ExpUBA35.366/2010 Res CD 2815/2010) and *Comité de Bioética, Fundación Huésped* (18/05/2009, Buenos Aires, Argentina). Both HIV-infected participants and healthy donors provided written informed consents accepting to participate in this study. 

### 2.3. Samples

Blood samples were collected from study participants at enrollment (baseline sample) and at 6 and 12 months post-presumed date of infection. Whole blood was centrifuged to separate plasma and stored at −80 °C until use. Peripheral blood mononuclear cells (PBMCs) were isolated by Ficoll–Hypaque density gradient centrifugation (GE Healthcare, Little Chalfont, UK) and cryopreserved for subsequent functional assays.

### 2.4. HIV-1 Viral Load, CD4^+^ T-cell Count and Immune Activation

Plasma VL was determined by branched-DNA assay (bDNA, Versant HIV-1 RNA 3.0 assay, Siemmens Healthcare, Erlangen, Germany). CD4^+^ T-cell count was determined by flow cytometry double platform (BD FACSCanto, BD Biosciences, San Jose, CA, USA). CD4^+^ and CD8^+^ lymphocyte activation was analyzed on thawed and over-night rested PBMCs by flow cytometry. Cells were stained for 30 min at 4 °C with LIVE/DEAD Fixable NEAR-IR (Life Technologies, Carlsbad, CA, USA) to exclude dead cells, and with the following fluorochrome-conjugated antibodies (all of them from BD Biosciences, San Jose, CA, USA): anti-HLA- antigen D Related (DR)- Fluorescein isothiocyanate (FITC), anti-CD4-Peridinin Chlorophyll Protein Complex (PerCP), anti-CD38- Allophycocyanin (APC), anti-CD3-Phycoerythrin-Cyanin7 (PeCy7) and anti-CD8-Phycoerythrin (PE). Cells were acquired in a BD FACSCanto flow cytometer. Data acquisition and analysis was performed using the BD FACSDiva v8.0.1 software (BD Biosciences, San Jose, CA, USA). Initial gating was performed on living lymphocytes followed by gating on CD3^+^CD4^+^ or CD3^+^CD8^+^ events. Isotype-matched FITC- and APC-conjugated non-specific antibodies were used in each sample to accurately set HLA-DR and CD38 negative populations.

### 2.5. Human Leukocyte Antigen (HLA) and CCR5 Genotyping

HLA class I A and B typing was performed using an in-house protocol consisting in PCR amplification, nucleotide sequencing with nested primers and web-based sequence interpretation. CCR5-Δ32 deletion was identified by differences in PCR product size. Single Nucleotide Polymorphisms (SNPs) of the CCR5 gene corresponding to positions 29, 208, 627, 630, 676 and 927 were determined with Site Directed Mutagenesis-PCR-Restriction Fragment Length Polymorphism (SDM-PCR-RFLP) assay. All procedures were previously described in Coloccini et al. [30].

### 2.6. HIV-Specific Cellular Immune Responses

The magnitude and specificity of the HIV-specific cellular immune response were screened by interferon (IFN)-γ Enzyme-Linked ImmunoSpot Assay (ELISPOT) in baseline samples as described previously [28,34].

### 2.7. Quantitation of Soluble Plasma Factors

Simultaneous determination of the following 39 cytokines and chemokines was performed using Luminex technology (MILLIPLEX MAP Human Cytokine/Chemokine, Merck Millipore, Billerica, MA, USA) in baseline samples: Endothelial growth factor (EGF), Eotaxin, fibroblast growth factor (FGF)-2, Fms-like tyrosine kinase (Flt)-3 Ligand, Fractalkine, granulocyte colony-stimulating factor (G-CSF), granulocyte-monocyte (GM)-CSF, GRO, IFN-α2, IFN-γ, interleukin (IL)-1α, IL-1β, IL-1rα, IL-2, IL-3, IL-4, IL-5, IL-6, IL-7, IL-8, IL-9, IL-10, IL-12 (p40), IL-12 (p70), IL-13, IL-15, IL-17, interferon gamma-induced protein (IP)-10, monocyte chemoattractant protein (MCP)-1, MCP-3, macrophage derived chemokine (MDC) (C-C motif chemokine (CCL)22), macrophage inflammatory protein (MIP)-1α, MIP-1β, soluble CD40 ligand (sCD40L), soluble IL 2 receptor alpha (sIL-2Rα), tumor growth factor (TGF)-α, tumor necrosis factor (TNF)-α, TNF-β, and vascular endothelial growth factor (VEGF). Samples were processed and analyzed as described elsewhere [35]. Plasma lipopolysaccharide (LPS) quantitation was performed using the Limulus Amebocyte Lysate (LAL) assay (QCL-1000, Lonza, Walkersville, MD, USA).

### 2.8. Definitions and Data Analysis

**Figure 1:**
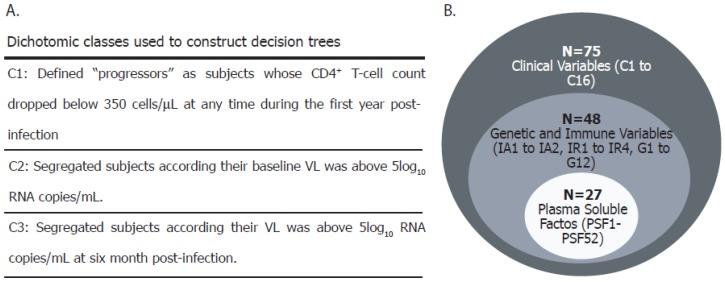
(**A**) Definition of the three classes (C1, C2 and C3) used in this study to segregate subjects into “progressors” and “non-progressors” or in relation to their ability to control viral replication. These classes were used to construct decision trees. (**B**) The whole database was subdivided into three self-including parts, i.e., the small database (*N* = 27), the intermediate base (*N* = 48) and the entire base (*N* = 75). The dataset used in each analysis is indicated in the text. CD4^+^ and CD8^+^ T-cell counts and viral load (VL) were determined in all subjects. Additionally, Human Leukocyte Antigen (HLA) and C-C chemokine receptor type 5 (CCR5) genotyping (and the corresponding genetic scores (GS, Appendix A)), HIV-specific immune responses and immune activation were determined in a subset of 48 subjects. Finally, 40 plasma soluble factors (39 cytokines and chemokines plus lipopolysaccharide (LPS) were quantified in a smaller group (*N* = 27). The values obtained for each soluble factor were used individually but also additive scores were constructed (CS, cytokine scores, Appendix A variables plasma soluble factor (PSF)41 to PSF52). IA: Immune activation; IR: Immune response.

The presumed date of infection was estimated as 14 days before the onset of symptoms or, in asymptomatic subjects, as the midpoint between the last negative and the first positive test or one month before the date of the indeterminate or negative Western blot assay [27]. Three dichotomic classes were constructed to segregate subjects according to their rate of disease progression over the first year postinfection or their capacity to control viral replication: C1 (defined on immunological criteria), C2 and C3 (defined on virological criteria). In C1, subjects were segregated according to whether their CD4^+^ T-cell count dropped below 350 cells/μL at any time during the first year postinfection (“progressors”), or not (“non-progressors”) [27,28,29,31,36]. The 350 cells/μL-endpoint was chosen based on the national and international recommendations for HAART initiation by the year 2010, when most of these individuals were already enrolled. C2 segregated subjects according to whether they had >100,000 baseline HIV RNA copies/mL plasma or not, based on previous observations from the same cohort [27]. Similarly, C3 segregated subjects according to whether they had >100,000 HIV RNA copies/mL plasma or not at the 6-month sample (Figure 1A). The CD4 slope was calculated for each subject as the slope of the best-fit line obtained by linear regression of CD4^+^ T-cell counts during the first year of infection and was represented as the CD4^+^ T-cell count change per day (cells/µL/day). Additive scores were created for each subject to compile host genetic information, as previously described by our group [30]. Similarly, scores based on cytokine data were created. Construction of genetic and cytokine-based scores is described in Appendix A, where all variables evaluated in this study are defined. For certain analyses, the whole database was subdivided in three self-including parts, i.e., the small database (*N* = 27), the intermediate database (*N* = 48) and the whole database (*N* = 75) (Figure 1B).

Statistical analyses were performed using GraphPad Prism 7 (GraphPad Software Inc., La Jolla, CA, USA) and IBM Corp. Released 2013, IBM SPSS Statistics for Windows, Version 22.0. Armonk, NY: IBM Corp. All data were analyzed using nonparametric statistics, unless otherwise stated. All *p*-values were two-sided and considered to be statistically significant when *p* < 0.05. For correlations involving plasma cytokine levels, *p*-values were adjusted for multiple comparisons using a false discovery rate (FDR) procedure, according to the Benjamini and Hochberg method, with R Project software v. 3.4.1 (R Foundation for Statistical Computing, Vienna, Austria). Adjusted *p*-values were considered significant when less than 0.1. Finally, data was analyzed by machine learning methods. Variable hierarchization was performed by Weka [37] (http://www.cs.waikato.ac.nz/ml/weka/v.3.7) using correlation-based feature selection and J48 decision tree. Pictures of decision trees were made using KNIME Analytics Platform (http://www.knime.com v3.4). Only trees with accuracy (of classification) and κ-Cohen [38] values above 0.7 and 0.6, respectively, were reported as significant.

## 3. Results

### 3.1. Cohort Description

**Figure 2:**
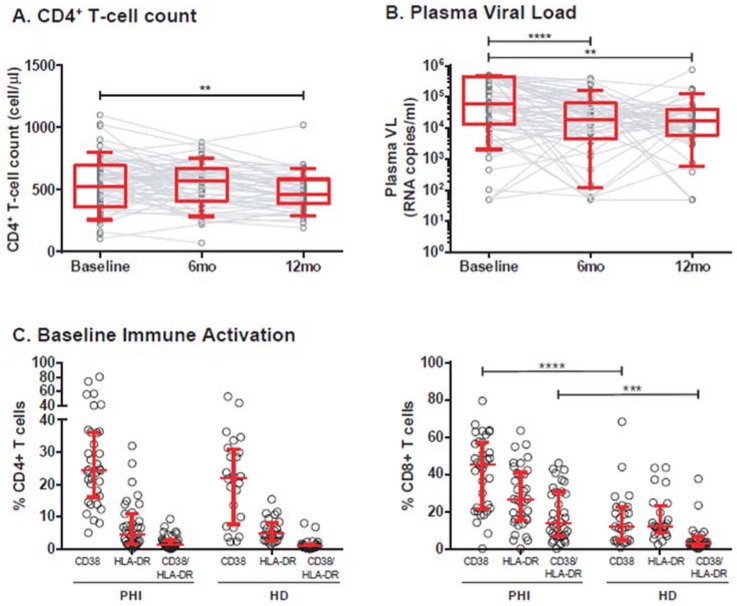
CD4^+^ T-cell counts, plasma viral load (VL), and immune activation of enrolled subjects. All samples were obtained as long as the subjects remained treatment naïve. Longitudinal determination of CD4^+^ T-cell counts (**A**); and plasma viral load (**B**) are shown (Baseline = enrollment sample, 6 and 12 months postinfection). Dots represent data from individual subjects and lines join matched values for each subject. Boxes represent the interquartile 25–75% range (IQR25–75) and whiskers extend from 10th to 90th percentiles. Horizontal lines within boxes represent the median. Immune activation (**C**) was evaluated at baseline as the percentage of CD38^+^, HLA-DR^+^ or CD38^+^/HLA-DR^+^ CD4^+^ (left panel) or CD8^+^ (right panel) T-cells. Dots represent data from individual subjects. Median and IQR25-75 are shown in red. In A and B, *p*-values were calculated using Wilcoxon test (baseline versus 6-month or 12-month samples). In C, *p*-values were calculated using Kruskal–Wallis test followed by Dunn’s post-test to compare preselected pairs of datasets. PHI: Primary HIV infection cohort. HD: Healthy donors. Asterisks denote *p*-values as follows: ** *p* < 0.01, *** *p* < 0.005, **** *p* <0.0001.

A total of 75 recently-infected HIV positive (HIV^+^) subjects were enrolled. The summary of subject´s characteristics is shown in Table 1. Eighty percent of enrolled subjects were identified on the bases of presenting symptoms compatible to acute retroviral syndrome. Baseline samples were obtained at a median of 75 days after the presumed date of infection. Most of the subjects were on Fiebig V at enrollment. Medical and laboratory evaluation (CD4^+^ T-cell count and VL) were performed at enrollment (baseline sample) and through one year. For the purposes of this study, data were recorded as long as subjects remained off-ART. Thus, sample size dropped to *N* = 59 and *N* = 46 at 6 and 12 months postinfection, respectively. CD4^+^ and CD8^+^ T-cell counts and HIV^+^ plasma viral load were determined longitudinally in the whole set of subjects (Table 1, Figure 2A,B). CD4^+^ T-cell count was significantly lower at the 12-month sample compared to baseline (*p* = 0.001). Although CD4^+^ T-cell counts were not determined in healthy donors (HD) enrolled for this study, unpublished data from our group indicate that median CD4^+^ T-cell counts for a similar adult population from our country (*N* = 118) is 834 cells/μL (IQR25–75% = 627–1080). This is significantly higher (Mann–Whitney test *p* < 0.0001) compared to the baseline sample from the HIV^+^ subjects thus reflecting the early attrition of the CD4^+^ T-cell subset which is characteristic of HIV infection. On the other hand, baseline VL was significantly higher than 6-month and 12-month VLs (*p* < 0.0001 and *p* = 0.0026, respectively). However, no difference was observed between 6-month and 12-month VLs, indicating that a set-point was already established. Baseline immune activation was determined in a subset of 48 subjects. At the CD8^+^ T-cell compartment, it was significantly higher than in healthy donors evidenced by higher percentages of CD8^+^ T-cells expressing CD38 (*p* < 0.001) and CD38/HLA-DR (*p* = 0.0006) (Table 1, Figure 2C).

A total of 88 variables, including those described in the preceding paragraph were recorded in enrolled subject (Appendix A). Due to technical limitations, some of these parameters were determined in smaller cohort subsets (see Figure 1). Thus, plasma LPS, cytokines and chemokines were determined in 27 subjects. These data were used to construct 12 cytokine-based scores (CS, Appendix A variables PSF41 to PSF52). Similarly, immune activation, HLA and CCR5 genotypes, genetic scores (GS, Appendix A variables G1 to G10) as well as magnitude and percentages of Nef- and Gag-specific immune cells were determined in 48 subjects. Gender ratio and median age at enrollment were similar across the three datasets.

### 3.2. Association of Individual Parameters with Disease Progression

We have previously described certain variables that, individually, were associated with disease progression in this cohort. For the purpose of this study we chose to include the following variables in this new analysis: immune activation because its level early after infection was associated with disease progression [31] (variables IA1-IA6, see Appendix A), HIV-specific T-cell specificity because we have shown that early Gag immunodominance was associated with slow rates of disease progression [28] (magnitude and percentages of Nef and Gag-specific T-cells; variables IR1-IR4), and genetic scores built based on subject´s HLA and CCR5 genotypes because one score was previously associated with lower baseline VL in this cohort [30] (variables G1-G8). These variables were chosen from the whole set of variables that we investigated in this cohort, based on their strong association with disease progression, and for their practicability to be measured in case it could be translated into the clinical setting. In addition to this, cytokines, chemokines and LPS were evaluated in plasma from enrolled subjects at baseline sample. Since the behavior of these variables in this cohort has not been described previously, we first analyzed the expression of these factors in comparison to HD and its association with markers of disease progression.

**Figure 3:**
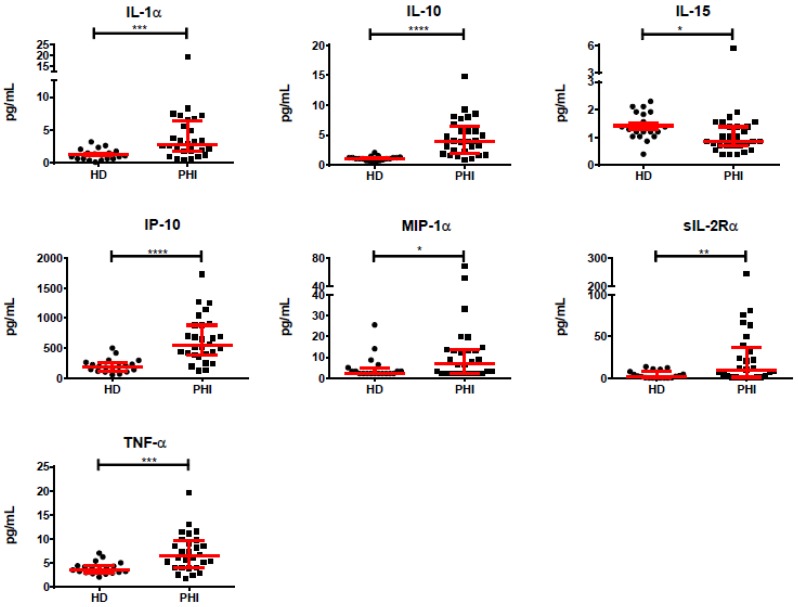
Plasma levels of IL-1α, IL-10, IL-15, IP-10, MIP-1α, sIL-2Rα, and TNF-α in samples obtained at enrollment of recently infected HIV^+^ subjects (PHI, baseline samples) and healthy donors (HD). Dots represent data from individual subjects. Median and interquartile ranges (IQR25–75) are shown in red. *p*-values were calculated using Mann–Whitney test. Asterisks denote *p*-values as follows: * *p* < 0.05, ** *p* < 0.01, *** *p* < 0.005, **** *p* < 0.0001. After false discovery rate (FDR) adjustment, all but macrophage inflammatory protein (MIP)-1α remained significantly different.

Out of the 39 cytokines and chemokines evaluated in plasma from enrolled subjects (*N* = 27), six were significantly increased during PHI compared to HD: IL-1α, IL-10, IP-10, MIP-1α, sIL-2Rα, and TNF-α. The highest increase was observed for sIL-2Rα which was seven-fold higher in PHI compared to HD. This was followed by IL-1α, IL-10, MIP-1α, and IP-10 which were three times elevated in PHI compared to HD, and TNF-α which was 1.7 times elevated in PHI. All but MIP-1α remained significantly elevated after adjustment for multiple comparisons (FDR procedure). IL-15 was significantly lower in PHI compared to HD (around 30% lower), even after FDR correction (Figure 3). Then, we aimed to study interconnection of cytokines both in HD and in PHI, following the rational proposed by Huang et al. [39]. For this, correlation analyses were performed among all cytokines in both groups. In HD, 144 out of 647 correlations evaluated were statistically significant (22.7%) while this ratio was 252/740 (34%) in PHI. Of those significant correlations, the r coefficients were significantly higher in HD (median = 0.7335, IQR25–75 = 0.6755–0.8215) compared to PHI (median = 0.4935 IQR25–75 = 0.435–0.555, *p* < 0.0001). Compared to HD, 68 significant correlations were maintained in PHI, 71 significant correlations were lost, and 180 new significant correlations emerged. This evidenced a rearrangement of the cytokine network during PHI, compared to HD.

**Figure 4:**
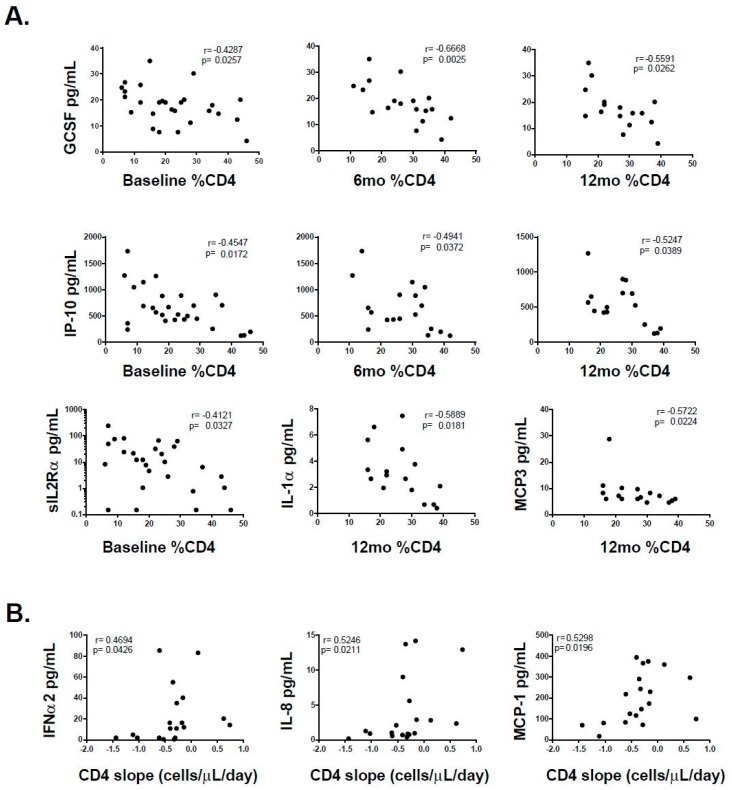
(**A**) Correlations between plasma level of denoted cytokines and chemokines (evaluated at baseline samples) versus percentages of CD4^+^ T-cells evaluated at the denoted time-points. (**B**) Correlations between the plasma level of denoted cytokines and chemokines (evaluated at baseline samples) versus daily CD4^+^ T-cell count decay rate (CD4 slope, cells/μL/day). Dots represent data from individual subjects. In the inset, r (upper line) and p (lower line) values correspond to Spearman’s test. After correction for multiple comparisons (FDR procedure) was applied, none of these correlations remained statistically significant.

**Figure 5:**
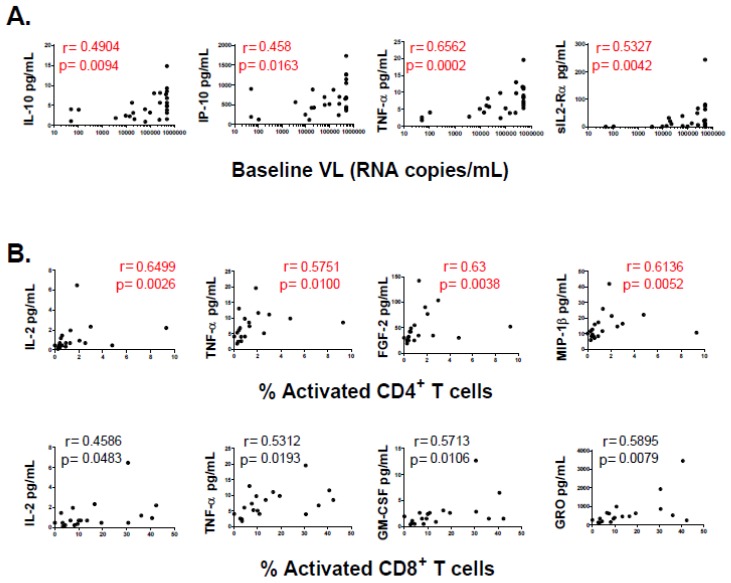
Correlations between plasma level of denoted cytokines and chemokines (evaluated at baseline samples) versus: plasma viral load (VL) (**A**); and baseline immune activation (**B**) (percentages of CD38^+^/HLA-DR^+^ CD4^+^ (upper panels) and CD8^+^ (lower panels) T-cells). Dots represent data from individual subjects. In the inset, r (upper line) and p (lower line) values correspond to Spearman’s test. After correction for multiple comparisons (FDR procedure) was applied, only those correlations shown in red remained statistically significant.

Then, the relation of each cytokine and chemokine with CD4^+^ T-cell counts, plasma VL and immune activation were determined. Baseline plasma levels of G-CSF and IP-10 inversely correlated with baseline, 6-month and 12-month CD4^+^ T-cell percentages (%CD4^+^ T-cells). Similarly, baseline plasma sIL-2Rα inversely correlated with baseline %CD4^+^ T-cells while baseline plasma IL-1α and MCP-3 inversely correlated with 12-month %CD4^+^ T-cells. In the same line, baseline plasma IFNα2, IL-8, MCP-1 directly correlated with CD4 slope (Figure 4). However, all these associations lost statistical significance after FDR adjustment. On the other hand, baseline plasma IL-10, IP-10, TNF-α and sIL-2Rα directly correlated with baseline VL and remained significant after adjustment (Figure 5). However, no statistically significant association was found between cytokine and chemokine levels and 6-month or 12-month VL. Of note, subjects with over-limited baseline VLs (>500,000 RNA copies/mL) were included in the analysis by setting the corresponding values at 500,000. The same analysis was repeated excluding these values and all correlations remained statistically significant except for IP-10. However, it should be noted that IP-10 is consistently found in the bibliography directly associated with VL (See the discussion section). Thus, this lack of correlation is most likely the result of reducing significantly the sample size (from 27 to 18) and the presence of one outlier. Finally, correlations between these plasma molecules and baseline immune activation (defined as percentages of CD4^+^CD38^+^HLA-DR^+^ and CD8^+^CD38^+^HLA-DR^+^ T-cells) were studied. Baseline IL-2, TNF-α, FGF-2 and MIP-1β directly correlated with percentages of activated CD4^+^ T-cells, while IL-2, TNF-α, GM-CSF and GRO directly correlated with percentages of activated CD8^+^ T-cells (Figure 5). However, only those correlations involving percentages of activated CD4^+^ T-cells remained significant after FDR adjustment. In summary, a few cytokines and chemokines, most of them associated with a pro-inflammatory profile except for IL-10, were elevated in PHI compared to HD. In contrast, IL-15 was diminished. Some associations were found between baseline levels of these cytokines and CD4^+^ T-cell count along time and also with CD4^+^ T-cell decay rate (CD4 slope). Though, these correlations lost significance after correction, suggesting that they might not represent truly associations. Conversely, correlations between IL-10, IP-10, TNF-α and sIL-2Rα and VL remained significant but they were only associated with baseline VL and not with 6-month and 12-month VL, which suggests that these molecules may not have any predictive value over the course of infection but they would only be related to the level of concurrent viral replication. No significant correlation was found between baseline plasma LPS levels and CD4^+^ T-cell counts or plasma VL at any time-point.

### 3.3. Baseline CD4^+^ T-cell Count Was the Most Potent Variable to Distinguish “Progressors” from “Non-Progressors”

**Figure 6:**
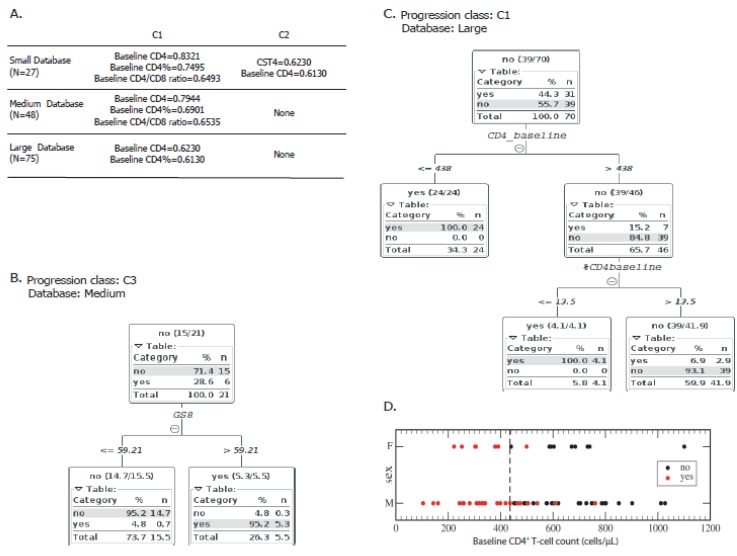
(**A**) Variable hierarchization using correlation based feature selection between classes C1 and C2 with all other individual variables as well as cytokine-based (CS) and genetic scores (GS), along with the three databases (small (*N* = 27), medium (*N* = 48), and large (*N* = 75)). Only strong correlations (>0.6) are shown. No correlation was found between the variables studied and C3. Decision trees were constructed to discriminate “progressors” from “non-progressors” as defined by: C3 (**B**); and C1 (**C**). To build the tree shown in (B), the clinical variables were not included in the analysis. (**D**) Baseline CD4^+^ T-cell counts of the individuals enrolled segregated as “progressors” (yes, red dots) and “non-progressors” (no, black dots) according to C1. The cut-off value as defined by the tree shown in C is depicted by the vertical dashed line. CST4: cytokine score CST4 (see Appendix A). GS8: genetic score 8 (see Appendix A). The number of instances considered in D and C was the result of the elimination of cases with missing class value.

Then, we aimed to weight the association of each variable with disease progression. For this purpose, three individual discrete dichotomic classes were constructed to segregate subjects according the criteria described in Materials and Methods section, and as shown in Figure 1A. In addition, for these analyses the whole database was subdivided into three self-including parts, i.e., the small database (*N* = 27), the intermediate database (*N* = 48) and the whole database (*N* = 75) (see Methods and Figure 1B). First, correlations between C1, C2 and C3 with all other individual variables as well as cytokine-based and genetic scores were studied using Weka correlation based feature selection along the three databases (Figure 6A). Strong correlations (*r* > 0.6) were mainly found with clinical parameters: baseline CD4^+^ T-cell counts, baseline CD4^+^ T-cell percentages and baseline CD4/CD8 ratios strongly correlated with C1 (*r* = 0.8321, 0.7495 and 0.6493, respectively). The r values correspond to correlations studied based on the small database (*N* = 27), although they remained above 0.6 when repeating the analyses with the intermediate database (0.7944, 0.6901 and 0.6535, respectively). However, only baseline CD4^+^ T-cell counts and baseline CD4^+^ T-cell percentages strongly correlated with class C1 when using the largest dataset (0.7210 and 0.62983, respectively). CST4 (Appendix A) and baseline CD4^+^ T-cell counts strongly correlated with C2 (*r* = 0.62307 and 0.6130, respectively) in the small database. However, the magnitude of the latter association was lost when using the bigger database. Surprisingly, no strong correlation was found when studying C3 in all the analyses. 

Then, with the aim of detecting the most predictive variables for infection progression, J48 decision trees were constructed using different sets of variables on the three self-including databases. Thus, for each database, trees were automatically generated using the whole set of variables (including genetic and cytokine-based scores), the intermediate subset of variables (all variables excluding the clinical dataset) and a small set of variables comprising only the cytokine-related variables. By using this methodology, none of the cytokine-related or immune (immune activation or immune responses) variables had good predictive value to distinguish between the groups of subjects segregated according to C1, C2 or C3. Only GS8 (Appendix A) could discriminate “high 6-month VL” from “low 6-month VL” as defined by C3 with good power (accuracy = 0.905, κ-Cohen = 0.767, Figure 6B), provided the clinical variables were not included in the analysis. The resulting tree had only one branch that split the feature GS8 with a cut-off value=59.21: most of the “6-month VL below 10^5^” subjects were separated into the ≤59.21 branch while most of the “6-month VL above 10^5^” subjects were separated into the >59.21 branch. When the clinical parameters were included in the dataset to build the trees, these variables always outcompeted all other variables including the cytokine and genetic scores, i.e., immune activation, HIV-specific immune responses, HLA haplotypes and soluble factors had lower discrimination power when compared to clinical parameters. Always, the clinical parameters were included in the analysis, and baseline CD4^+^ T-cell count was the most potent variable to distinguish “progressors” from “non-progressors” defined by C1 with a cut-off of 438 cells/μL in the *N* = 75 database (accuracy = 0.929, κ-Cohen = 0.853; Figure 6C,D). Here, most “progressors” and no “non-progressors” were separated into the ≤438 branch. The >438 branch contained 15% “progressors” and 85% “non-progressors” that could be separated into a subsequent branch by the baseline percentage of CD4^+^ T-cells.

## 4. Discussion

Progression of HIV infection is variable among individuals and the definition of disease progression biomarkers is still fundamental. Apart from CD4^+^ T-cell count and VL, several parameters individually showed associations with the rate of disease progression, as shown by our group and others [11,12,13,14,15,16,17,18,19,20,21,22,23,24,25,26,27,28,29,30,31]. Here, we took advantage of results found in our well characterized cohort of acute/early HIV infected subjects from Argentina. This cohort was fully enrolled in our country, and the vast majority of the subjects enrolled are native, which distinguishes this cohort from others from Africa, Europe and Asia. The analysis included a rich dataset of 16 clinical variables, six immune activation variables, four cellular immune response variables, 10 genetic variables and 52 variables related to plasma soluble factors. The behavior of some of these variables in this cohort, in particular their individual associations with disease progression, has been described elsewhere [27,28,29,30,31]. The aim of the study was to categorize their predictive potential using decision trees and to analyze their possible implementation in the clinical setting. 

The so-called “cytokine storm” occurring during the first weeks after HIV infection is a well-known phenomenon [18,39,40,41]. In line with this, plasma levels of IL-1α, IL-10, IP-10, sIL-2Rα, and TNF-α were significantly elevated (from 1.7 to 7 times) in the baseline samples from subjects enrolled in the cohort, compared to HD. The elevation of IL-1α, IP-10, sIL-2Rα, TNF-α may be indicating the activation of a pro-inflammatory response, not only to HIV-encoded pathogen-associated molecular patterns (PAMPs) but also to bacterial PAMPs exposed as a result of the gut-associated lymphoid tissue early disruption [1,42]. IL-10 elevation might be the result of an attempt to control the pro-inflammatory burst [18,43]. In addition, it depicts the activation of innate immunity effectors and also T-cells. On the other hand, a modest albeit significant reduction in IL-15 was observed. This cytokine play key roles in both innate and adaptive responses by participating in the expansion and differentiation of Natural Killer (NK) cells and also by contributing to the homeostasis of the memory T cell pool. Several reports indicated that IL-15 was elevated during HIV acute infection [18,40,44,45] while others reported no changes [15,16]. These discrepancies could be attributed to the fact that IL-15 peaks very fast after infection and then sharply decays [39,41,44]. Thus, sampling time after infection can significantly affect the results. Apart from the mere increment in the plasma concentration of cytokines, a more intricate relationship between all cytokines was observed in acute infection, compared to HD, evidenced by the rearrangement of correlations among the cytokines studied. This is in line with a recent report indicating that HIV imposes a new order on the cytokine network, which in turn could contribute to disease [39].

Correlation analysis between plasma cytokines in baseline samples revealed several associations with clinical parameters such as CD4^+^ T-cell count, viral load and immune activation either evaluated in the same or in subsequent samples. All correlations involving CD4^+^ T-cell count (including CD4 slope) and CD8^+^ T-cell activation lost significance after adjustment for multiple comparison. Conversely, IL-2, TNF-α, FGF-2 and MIP-1β remained significantly correlated with concurrent CD4^+^ T-cell activation. Noel et al. have reported that plasma levels of IP-10, sCD163, IL-6 and MCP1 directly correlated both with CD4^+^ and CD8^+^ T-cell activation (measured as percentage of CD38^+^HLA-DR^+^ cells) in a cohort of chronically-infected HIV controllers [46]. More relevant to our context, Liovat et al. reported that levels of IP-10, IL-18 and TGF-β1, measured early after infection, correlated with CD8^+^ T-cell activation measured at six months postinfection, but CD4^+^ T-cell activation was not evaluated [16]. The fact that associations (and the strength of those associations) between individual cytokines and immune activation differed whether the evaluation was performed in the CD4^+^ or the CD8^+^ T-cell compartment provides support to the notion that different forces (for instance, immune homeostasis or viral replication itself), could be driving cellular activation in each compartment [43]. Above all, the strongest correlations were those found between baseline levels of IL-10, IP-10, TNF-α and sIL-2Rα with baseline VL. Of note, these cytokines did not correlate with subsequent VL values suggesting that they might have no predictive value over VL course, at least in this cohort. Instead, these associations might be reflecting that the magnitude of the “cytokine storm” is directly or indirectly influenced by the magnitude of concurrent viral replication as suggested by others [15,18,39]. Of note, these cytokines belong to divergent families and, with the exception of sIL2Rα, they have also been associated previously with the magnitude of HIV replication during acute infection [15,16]. TNF-α is a potent effector of the antiviral immunity and also can increase viral replication by enhancing proviral transcription. Suppressor activity of IL-10 might help control immune-mediated damage during infection and also limits HIV-specific response favoring viral persistence. IP-10 (or C-X-C motif chemokine (CXCL)-10) is a chemokine induced by IFN-γ, which has been consistently found to be elevated in different stages of HIV infection, including acute/early infection, and it has been mostly associated with the worst-case scenarios in terms of disease progression [15,39,46,47,48,49,50,51]. In particular, two studies [16,52] identified IP-10 level during acute infection as a predictor of rapid disease progression. Although in this work baseline IP-10 correlated with concurrent VL, it failed to predict subsequent VL or discriminate progressors from non-progressors, as in the publications mentioned.

Finding an early marker of disease progression is not a new concept but it has been proven to be a very difficult task. For instance, Roberts et al. [15] have developed a model to predict the VL set point and also a risk score of progression, both based on measuring a panel of plasma cytokines early after infection. Those equations in our cohort were tested but the outcome was unsuccessful in terms of progression prediction. The cohort used by Roberts et al. included exclusively African women, and showed a high prevalence of sexually transmitted infections other than HIV. On the contrary, our cohort comprises Caucasian men mainly. This, together with other limitations of our study, which are mentioned below, could explain the negative results when trying to apply their model in our cohort. Beyond that, it does nothing but emphasize the difficulties when trying to find a universal marker of HIV disease progression. In the same line, Mahnke et al. [12] postulated immune activation (measured as %CD38^+^CD8^+^ T-cells), cell-associated VL (CAVL) and CD8^+^ T-cell phenotype (%CCR5^+^CD8^+^ T-cells) as early predictors of disease progression. While CAVL and %CCR5^+^CD8^+^ T-cells were not measured in this study, %CD38^+^CD8^+^ T-cells showed no potential as predictive factors. As in our study, HIV-specific CD8^+^ T-cell response has also been evaluated by Mahnke et al. as a possible predictor of disease progression and, in consonance with our findings, it only proved to be a weak predictor of progression over other factors.

Discordant results between this and previous studies could be explained by differences in the premises used to define disease progression, in the methodologies used to analyze the data and the time in which the baseline samples were obtained (in relation to infection date). In particular, one limitation of this study might be the wide variation in the timing of baseline sample collection and that most samples were obtained during the late acute/early infection. However, this could also be interpreted as a better reflection of the real life, since the already unusual detection of an acutely infected subject in clinics, most usually happens at Fiebig stage IV–VI. Thus, the results obtained using this kind of more “real” cohorts would be easier to translate into the real practice. Above all, the discrepancies and the difficulty in finding a reliable acute infection marker of disease progression most likely reflect the diversity and the extreme dynamics of the events that follow HIV infection.

Here, after analyzing 88 variables in a well-defined cohort of seroconverters and where disease progression (within the first year postinfection) was defined by several means, baseline CD4^+^ T-cell count emerged as the only variable able to predict rapid progression by machine learning methods. Many correlations between variables have been studied and several significant values were obtained. However, strong correlations do not necessarily imply predictive power over disease progression. Based on this, a two-way analysis was used: first correlations were used for filtering variables and then their predictive power was analyzed using the J48 algorithm which generates the decision trees. In an attempt to identify potential co-biomarkers of progression that may go along with CD4^+^ T-cell count, all possibilities of analysis were exhausted, i.e., including and excluding sets of data one by one. However, these efforts were unsuccessful. Interestingly, when CD4^+^ T-cell count was the only variable removed from the analysis, baseline VL appeared as the next variable with power to discern “progressors” from “non-progressors”. No other variables (neither immune nor genetic) have been able to displace the classical parameters used to date to monitor disease progression. Thus, our approach reaffirms the predictive power of CD4^+^ T-cell count (and the lack of power of the other variables) despite the scarce available data. Interestingly, the CD4^+^ T-cell count complies with most requirements needed to be a good biomarker [5]: its assessment is objective and precise with current methodologies, it is reliable, it is directly related to the disease mechanism, and, as particularly shown in this work, it is able to identify early events in the natural history of the disease. Importantly, rapid, reliable, and affordable point-of-care CD4 tests are being developed. This will allow its rapid determination not only in centralized institutions but also in peripheral areas which will, in turn, rapidly aid in decision making and intervention [53]. In addition, this result highlights the importance of CD4^+^ T-cell count for monitoring HIV infection even when there is an increasing trend to minimize its use at least in virally suppressed subjects [54,55]. Noteworthy, a significant proportion of subjects (19 out of 75, 25%) had low CD4^+^ T-cell counts (<350 cells/μL) already at baseline sample, as observed also in other cohorts [56]. This emphasizes the need for interventions aimed at detecting acutely-infected subjects and at linking them to care immediately. On the other end, high baseline CD4^+^ T-cell counts (>500 cells/µL) were observed even in subjects that progressed rapidly after infection. This provides further support to comply with current treatment guidelines, which suggest early ART initiation. 

Overall, our machine learning approach to tackling this problem was based on feature selection and decision trees. Feature selection allowed us to reduce the large number of available variables to significant ones in terms of correlation with the class. On the other hand, decision tree algorithm provides appropriate classifiers to work with scarce data and with readable outputs in a simple way. Thus, the use of feature selection and decision trees proved to be a valid methodology to weight putative biomarkers of disease progression following HIV infection. In the era of HIV cure strategies research, studies aimed at identifying biomarkers of post-treatment control are being encouraged. For instance, it is being increasingly clear that the VL magnitude reached during acute/early infection directly correlates with the reservoir size after cART is started [57,58]. In the same line, persistently high levels of pro-inflammatory cytokines after ART initiation might indicate low-level ongoing viral replication in anatomically privileged sites [59]. Altogether, elevated cytokines early after infection could impact both the seeding and the maintenance of the viral reservoir. Thus, it is tempting to hypothesize that the cytokines that were elevated during PHI in our cohort and particularly those that associated with higher baseline VL would serve as biomarkers of reservoir size after cART initiation. This would be an instrumental tool in the context of cure strategies. Thus, subsequent studies in this field using feature selection and decision trees are warrantied. 

## Figures and Tables

**Table 1:** Characteristics of HIV^+^ subjects enrolled in this study.

**Total sample size (number of individuals)**		75	
**Female:Male ratio**		1:3	
**Age at enrollment (years, median and IQR25–75%)**		30 (24–38)	
**Estimated time of infection at enrollment (days, median and IQR25–75%)**		75 (54–113)	
**Follow-up of Virologic and Immune Characteristics:**			
	**Baseline (*N* = 75)**	**6-month pi (*N* = 59)**	**12-month pi (*N* = 46)**
**VL (RNA copies/mL; median and IQR25–75%) ^a^**	61,045 (12,736–455,417)	18,951 (4298–62,739)	16,988 (5695–40,105)
**Log_10_VL (mean ± SD) ^a^**	4.6 ± 1	4 ± 1	4 ±0.89
**CD4^+^ T-cell count (cells/μL, median and IQR25–75%) ^b^**	525 (361–698)	571 (406–673)	464 (387–585)
**CD4/CD8 Ratio (Median and IQR25–75%)**	0.6 (0.32–0.83)	0.55 (0.34–0.8)	0.61 (0.39–0.93)
**CD4^+^ T-cell decay rate (cells/μL/day; median and IQR25–75%)**	−0.62 (−0.31 – −0.03)		
**Baseline Immune Activation (%cells, median and IQR25–75%) ^c^:**			
**%CD4^+^CD38^+^**	24.4 (16–36.2)		
**%CD4^+^HLA-DR^+^**	4.6 (1.5–11.01)		
**%CD4^+^CD38^+^HLA-DR^+^**	1.2 (0.47–2.8)		
**%CD8^+^CD38^+^**	45.2 (21.3–57.1)		
**%CD8^+^HLA-DR^+^**	26.5 (15.2–41)		
**%CD8^+^CD38^+^HLA-DR^+^**	13.8 (6.7–30.6)		

^a^ Versant HIV-1 RNA 3.0 assay, Siemens. Lower and upper detection limits are 50 and 500,000 RNA copies/mL, respectively (1.7log10 and 5.7log10); ^b^ Flow cytometry double platform, FACSCanto, BD Biosciences; ^c^ Immune activation was only evaluated at baseline samples by flow cytometry. IQR25–75: Interquartile range 25–75%. VL: Viral Load. pi: postinfection. SD: Standard deviation. IQR: interquartile range; HLA-DR: Human leukocyte antigen - antigen D Related.

## Appendix A

**Table A1:** List and description of variables evaluated in this study. In addition to individual variables, scores were constructed to compile host genetic (variables G1 to G10) and plasma soluble factor (PSF41 to PSF52) data for each subject.

Variable ID	Variable Name	Variable Description
C1	Baseline CD4	Absolute CD4^+^ T-cell count evaluated at enrollment
C2	Baseline %CD4	Percentage of CD4^+^ T-cells evaluated at enrollment
C3	Baseline CD4/CD8	CD4/CD8 ratio evaluated at enrollment
C4	6mo CD4	Absolute CD4^+^ T-cell count evaluated at 6 months postinfection
C5	6mo %CD4	Percentage of CD4^+^ T-cells evaluated at 6 months postinfection
C6	6mo CD4/CD8	CD4/CD8 ratio evaluated at 6 months postinfection
C7	12mo CD4	Absolute CD4^+^ T-cell count evaluated at 12 months postinfection
C8	12mo %CD4	Percentage of CD4^+^ T-cells evaluated at 12 months postinfection
C9	12mo CD4/CD8	CD4/CD8 ratio evaluated at 12 months postinfection
C10	CD4 Slope	Rate of CD4^+^ T-cell decay over the first year postinfection (cells/µL/day)
C11	Baseline VL	Plasma viral load evaluated at enrollment (RNA copies/mL)
C12	Baseline log_10_VL	Log_10_ plasma viral load evaluated at enrollment
C13	6mo VL	Plasma viral load evaluated at 6 months postinfection (RNA copies/mL)
C14	6mo log_10_VL	Log_10_ plasma viral load evaluated at 6 months postinfection
C15	12mo VL	Plasma viral load evaluated at 12 months postinfection (RNA copies/mL)
C16	12mo log_10_VL	Log_10_ plasma viral load evaluated at 12 months postinfection
IA1	CD4CD38	Percentage of CD4^+^ CD38^+^ T-cells evaluated at enrollment
IA2	CD4HLADR	Percentage of CD4^+^ HLA-DR^+^ T-cells evaluated at enrollment
IA3	CD4double	Percentage of CD4^+^ CD38^+^ HLA-DR^+^ T-cells evaluated at enrollment
IA4	CD8CD38	Percentage of CD8^+^ CD38^+^ T-cells evaluated at enrollment
IA5	CD8HLADR	Percentage of CD8^+^ HLA-DR^+^ T-cells evaluated at enrollment
IA6	CD8double	Percentage of CD8^+^ CD38^+^ HLA-DR^+^ T-cells evaluated at enrollment
IR1	%Nef response	Percentage of T-cell response directed to Nef (over total HIV-specific response) evaluated at enrollment by ELISPOT
IR2	Absolute Nef response	Magnitude of Nef-specific response evaluated at enrollment by ELISPOT (SFU/million PBMC)
IR3	%Gag response	Percentage of T-cell response directed to Gag (over total HIV-specific response) evaluated at enrollment by ELISPOT
IR4	Absolute Gag response	Magnitude of Gag-specific response evaluated at enrollment by ELISPOT (SFU/million PBMC)
G1	GS1	Additive genetic score constructed based on the presence or absence of certain HLA alleles, as described previously [30]. Alleles with a previous reported protective effect were added (+1), and risk alleles were subtracted (-1). Based on relevant bibliography, HLA-A*02, HLA-A*32, HLA-A*68, HLA-B*15, HLA-B*13, HLA-B*27, HLA-B*32, HLA-B*39, HLA-B*44, HLA-B*51 and HLA-B*57 were considered as protective. HLA-A*11, HLA-A*23, HLA-A*24, HLA-B*08, HLA-B*35, HLA-B*53, HLA-C*04 and HLA-C*07 were considered as deleterious. Other HLA alleles were considered as neutral (0). Heterozygosis for HLA was considered as protective (1) and homozygosis as deleterious (–1).
G2	GS3	Additive genetic score constructed based on the presence or absence of certain HLA alleles and CCR5 genotypes, as described previously [30]. HLA alleles associated with protection or risk were considered as in GS1. For CCR5 polymorphisms, Δ32 and CCR2-64I alleles were considered as protective. CCR5 genotypes HHC/HHF*2 and HHC/HHG*2 were considered as protective (+1), HHC/HHE, HHE/HHE and HHE/HHG*2 were considered as deleterious (–1), and the others as neutral (0).
G3	GS5	Additive genetic score constructed based on adding +1 when a protective allele was present and subtracting −1 when a risk allele was present. Protective and risk alleles were determined based on the odd ratio obtained for each allele in our cohort with a *p* < 0.05. HLA alleles with *p* > 0.05 were considered as neutral (0).
G4	GS6	Idem to GS5 but the cut-off level for significance was established at *p* < 0.03.
G5	GS7	Constructed by multiplying the odds ratio corresponding to the 6-month CD4^+^ T-cell count for each of the 6 HLA alleles (A, B and C) of each individual.
G6	GS8	Constructed by multiplying the odds ratio corresponding to the 6-month VL for each of the 6 HLA alleles (A, B and C) of each individual.
G7	GS9	Constructed by multiplying the odds ratio corresponding to the 6-month CD4^+^ T-cell count for each of the 2 CCR5 haplotypes of each individual.
G8	GS10	Constructed by multiplying the odds ratio corresponding to the 6-month VL for each of the 2 CCR5 haplotypes of each individual.
G9	GS11	Constructed by multiplying the odds ratio corresponding to the 6-month CD4^+^ T-cell count for each of the 6 HLA alleles (A, B and C) and the 2 CCR5 haplotypes of each individual.
G10	GS12	Constructed by multiplying the odds ratio corresponding to the 6-month VL for each of the 6 HLA alleles (A, B and C) and the 2 CCR5 haplotypes of each individual.
PSF1	EGF	Plasma level of EGF (Endothelial growth factor) evaluated at enrollment by Luminex (pg/mL)
PSF2	Eotaxin	Plasma level of Eotaxin evaluated at enrollment by Luminex (pg/mL)
PSF3	FGF2	Plasma level of FGF-2 (fibroblast growth factor 2) evaluated at enrollment by Luminex (pg/mL)
PSF4	Flt3Ligand	Plasma level of Flt-3 (Fms-like tyrosine kinase 3) ligand evaluated at enrollment by Luminex (pg/mL)
PSF5	Fractalkine	Plasma level of Fractalkine evaluated at enrollment by Luminex (pg/mL)
PSF6	GCSF	Plasma level of G-CSF (granulocyte colony-stimulating factor) evaluated at enrollment by Luminex (pg/mL)
PSF7	GMCSF	Plasma level of GM-CSF (granulocyte monocyte colony-stimulating factor) evaluated at enrollment by Luminex (pg/mL)
PSF8	GRO	Plasma level of GRO evaluated at enrollment by Luminex (pg/mL)
PSF9	IFN-α2	Plasma level of IFN-α2 (interferon alpha 2) evaluated at enrollment by Luminex (pg/mL)
PSF10	IFN-γ	Plasma level of IFN-γ (interferon gamma) evaluated at enrollment by Luminex (pg/mL)
PSF11	IL1α	Plasma level of IL-1α (interleukin 1 alpha) evaluated at enrollment by Luminex (pg/mL)
PSF12	IL1β	Plasma level of IL-1β (interleukin 1 beta) evaluated at enrollment by Luminex (pg/mL)
PSF13	IL1ra	Plasma level of IL-1ra (interleukin 1 receptor antagonist) evaluated at enrollment by Luminex (pg/mL)
PSF14	IL2	Plasma level of IL-2 (interleukin 2) evaluated at enrollment by Luminex (pg/mL)
PSF15	IL3	Plasma level of IL-3 (interleukin 3) evaluated at enrollment by Luminex (pg/mL)
PSF16	IL4	Plasma level of IL-4 (interleukin 4) evaluated at enrollment by Luminex (pg/mL)
PSF17	IL5	Plasma level of IL-5 (interleukin 5) evaluated at enrollment by Luminex (pg/mL)
PSF18	IL6	Plasma level of IL-6 (interleukin 6) evaluated at enrollment by Luminex (pg/mL)
PSF19	IL7	Plasma level of IL-7 (interleukin 7) evaluated at enrollment by Luminex (pg/mL)
PSF20	IL8	Plasma level of IL-8 (interleukin 8) evaluated at enrollment by Luminex (pg/mL)
PSF21	IL9	Plasma level of IL-9 (interleukin 9) evaluated at enrollment by Luminex (pg/mL)
PSF22	IL10	Plasma level of IL-10 (interleukin 10) evaluated at enrollment by Luminex (pg/mL)
PSF23	IL12p40	Plasma level of IL-12p40 (interleukin 12 subunit p40) evaluated at enrollment by Luminex (pg/mL)
PSF24	IL12p70	Plasma level of IL-12p70 (interleukin 12) evaluated at enrollment by Luminex (pg/mL)
PSF25	IL13	Plasma level of IL-13 (interleukin 13) evaluated at enrollment by Luminex (pg/mL)
PSF26	IL15	Plasma level of IL-15 (interleukin 15) evaluated at enrollment by Luminex (pg/mL)
PSF27	IL17	Plasma level of IL-17 (interleukin 17) evaluated at enrollment by Luminex (pg/mL)
PSF28	IP10	Plasma level of IP10 (interferon gamma-induced protein 10, CXCL10) evaluated at enrollment by Luminex (pg/mL)
PSF29	MCP1	Plasma level of MCP1 (monocyte chemoattractant protein 1) evaluated at enrollment by Luminex (pg/mL)
PSF30	MCP3	Plasma level of MCP3 (monocyte chemoattractant protein 3) evaluated at enrollment by Luminex (pg/mL)
PSF31	MDC	Plasma level of MDC (macrophage derived chemokine, CCL22) evaluated at enrollment by Luminex (pg/mL)
PSF32	MIP1α	Plasma level of MIP-1α (macrophage inflammatory protein 1 alpha) evaluated at enrollment by Luminex (pg/mL)
PSF33	MIP1β	Plasma level of MIP-1β (macrophage inflammatory protein 1 beta) evaluated at enrollment by Luminex (pg/mL)
PSF34	sCD40L	Plasma level of sCD40L (soluble CD40 ligand) evaluated at enrollment by Luminex (pg/mL)
PSF35	sIL2Rα	Plasma level of sIL-2Rα (soluble interleukin 2 receptor alpha) evaluated at enrollment by Luminex (pg/mL)
PSF36	TGFα	Plasma level of TGF-α (tumor growth factor alpha) evaluated at enrollment by Luminex (pg/mL)
PSF37	TNFα	Plasma level of TNF-α (tumor necrosis factor alpha) evaluated at enrollment by Luminex (pg/mL)
PSF38	TNFβ	Plasma level of TNF-β (tumor necrosis factor beta) evaluated at enrollment by Luminex (pg/mL)
PSF39	VEGF	Plasma level of VEGF (vascular endothelial growth factor) evaluated at enrollment by Luminex (pg/mL)
PSF40	LPS	Plasma level of LPS (lipopolysaccharide) evaluated at enrollment by Lal assay (EU/mL)
PSF41	CSVL1	Cytokines that significantly correlated with baseline VL were considered (sIL-2Rα, TNF-α, IP-10 and IL-10) to construct an additive score based on 1s and −1s. If the cytokine value of the subject was above 75% IQR corresponding to the group of healthy donors (HD), then this cytokine was assigned a value of 1. If the value was below 25% IQR corresponding to HD, it was assigned a value of −1. If the value was within the range IQR25–75% corresponding to HD, it was assigned a value of 0.
PSF42	CSCD41	Cytokines that significantly correlated with baseline CD4^+^ T-cell counts were considered (sIL-2Rα, IP-10 and G-CSF) to construct an additive score based on 1s and −1s. If the cytokine value of the subject was above 75% IQR corresponding to the group of healthy donors (HD), then this cytokine was assigned a value of −1. If the value was below 25% IQR corresponding to HD, it was assigned a value of 1. If the value was within the range IQR25–75% corresponding to HD, it was assigned a value of 0.
PSF43	CST1	Score defined as the arithmetic sum of CSVL1 + CSCD41.
PSF44	CSVL2	Cytokines that significantly correlated with baseline VL were considered (sIL-2Rα, TNF-α, IP-10 and IL-10) to construct an additive score based on adding the corresponding Spearman’s R values. If the cytokine value of the subject was above 75% IQR corresponding to the group of healthy donors (HD), then the Spearman´s R value corresponding to that cytokine was added to the score. If the value was below 25% IQR corresponding to HD, the Spearman´s R value corresponding to that cytokine was subtracted from the score. If the value was within the range IQR25–75% corresponding to HD, it was assigned a value of 0.
PSF45	CSCD42	Cytokines that significantly correlated with baseline CD4^+^ T-cell counts were considered (sIL-2Rα, IP-10 and G-CSF) to construct an additive score based on adding the corresponding Spearman’s R values. If the cytokine value of the subject was above 75% IQR corresponding to the group of healthy donors (HD), then the Spearman´s R value corresponding to that cytokine was subtracted from the score. If the value was below 25% IQR corresponding to HD, the Spearman´s R value corresponding to that cytokine was added to the score. If the value was within the range IQR25–75% corresponding to HD, it was assigned a value of 0.
PSF46	CST2	Score defined as the arithmetic sum of CSVL2 + CSCD42
PSF47	CSVL3	Cytokines that significantly correlated with baseline VL were considered (sIL-2Rα, TNF-α, IP-10 and IL-10) to construct an additive score based on normalizing the value of each of these cytokines over the cytokine median of the PHI group and multiplying this adjusted value by the corresponding Spearman´s R.
PSF48	CSCD43	Cytokines that significantly correlated with baseline CD4^+^ T-cell counts were considered (sIL-2Rα, IP-10 and G-CSF) to construct an additive score based on normalizing the value of each of these cytokines over the cytokine median of the PHI group and multiplying this adjusted value by the corresponding Spearman´s R.
PSF49	CST3	Score defined as the arithmetic sum of CSVL3 + CSCD43.
PSF50	CSVL4	Cytokines that significantly correlated with baseline VL were considered (sIL-2Rα, TNF-α, IP-10 and IL-10) to construct an additive score based on multiplying the log_10_ value of each of these cytokines by the corresponding Spearman´s R.
PSF51	CSCD44	Cytokines that significantly correlated with baseline CD4^+^ T-cell counts were considered (sIL-2Rα, IP-10 and G-CSF) to construct an additive score based on normalizing the log_10_ value of each of these cytokines by the corresponding Spearman´s R
PSF52	CST4	It was defined as the arithmetic sum of CSVL4 + CSCD44
